# LDL-Cholesterol and Platelets: Insights into Their Interactions in Atherosclerosis

**DOI:** 10.3390/life11010039

**Published:** 2021-01-11

**Authors:** Aleksandra Gąsecka, Sylwester Rogula, Łukasz Szarpak, Krzysztof J. Filipiak

**Affiliations:** 1 Department of Cardiology, Medical University of Warsaw, 02-091 Warsaw, Poland; sylwesterrogula@o2.pl (S.R.); krzysztof.filipiak@wum.edu.pl (K.J.F.); 2 Bialystok Oncology Center, 15-027, Bialystok, Poland; l.szarpak@fmcgroup.org; 3 Maria Sklodowska-Curie Medical Academy in Warsaw, 03-411 Warsaw, Poland

**Keywords:** LDL-cholesterol, platelets, extracellular vesicles, atherosclerosis, treatment

## Abstract

Atherosclerosis and its complications, including acute coronary syndromes, are the major cause of death worldwide. The two most important pathophysiological mechanisms underlying atherosclerosis include increased platelet activation and increased low-density lipoproteins (LDL) concentration. In contrast to LDL, oxidized (ox)-LDL have direct pro-thrombotic properties by functional interactions with platelets, leading to platelet activation and favoring thrombus formation. In this review, we summarize the currently available evidence on the interactions between LDL-cholesterol and platelets, which are based on (i) the presence of ox-LDL-binding sites on platelets, (ii) generation of ox-LDL by platelets and (iii) the role of activated platelets and ox-LDL in atherosclerosis. In addition, we elaborate on the clinical implications of these interactions, including development of the new therapeutic possibilities. The ability to understand and modulate mechanisms governing interactions between LDL-cholesterol and platelets may offer new treatment strategies for atherosclerosis prevention.

## 1. Introduction

Atherosclerosis and its complications, including acute coronary syndromes, are the major cause of death worldwide [1]. New insights into the mechanisms of atherosclerosis development and progression, which allow us to develop novel treatment strategies, are of utmost importance both from the health and economic viewpoints [2]. Pathologically, atherosclerosis is a chronic inflammation of the vessel wall due to factors that disturb the endothelium-dependent regulation of the vascular tone by nitric oxide (NO) formed by the constitutive endothelial nitric oxide synthase [3]. Impaired NO release together with a local enhanced NO degradation by reactive oxygen species leads to the imbalance between vasoconstriction and vasodilation and initiates numerous mechanisms that promote or exacerbate atherosclerosis, including increased endothelial permeability, leukocyte adhesion and generation of cytokines [4]. Among these various mechanisms, the interactions between platelets and low-density lipoproteins (LDL) are of particular interest for this review [5]. Platelets are involved both in the initiation of the vascular inflammatory in course of atherosclerosis, and promote thrombus formation after the atherosclerotic plaque rupture [6]. Activated platelets adhere not only to the damaged endothelium, but also to the intact or minimally altered endothelium, thus aggravating inflammatory and thrombotic processes [7]. LDL, in turn, participate in the early phase of atherosclerosis by recruiting inflammatory cells to the sub-endothelium and eliciting prothrombotic changes on the endothelial cell surface [8]. Several cells including platelets, endothelial cells and macrophages are involved in the process of oxidized (ox)-LDL formation [9,10]. Platelets are traditionally not defined as cells, as they are fragments of the cytoplasm of megakaryocytes and have no nucleus. However, some authors justify that platelets might be perceived as immune cells in the context of atherosclerosis development due to their numerous interactions with other cells [11]. Therefore, in this review, we define platelets as cells, bearing in mind that there is a discussion on this topic in the literature [11].

Ox-LDL are defined as “circulating LDL-derived particle which have peroxides or their degradation products associated with the LDL particle” [12]. In contrast to LDL, ox-LDL have direct pro-thrombotic properties by functional interactions with platelets, leading to platelet activation [13,14] and favouring thrombus formation [15]. In this review, we summarize the currently available evidence on the interactions between LDL-cholesterol and platelets, which are based on (i) the presence of LDL-binding sites on platelets, (ii) generation of ox-LDL by platelets and (iii) the role of activated platelets and ox-LDL in atherosclerosis. In addition, we elaborate on the clinical implications of these interactions, including development of the new therapeutic possibilities.

## 2. LDL-Binding Sites on Platelets

Ox-LDL activate platelets via interaction with two types of platelet receptors: cluster of differentiation (CD) 36 and lectin-like oxidized low-density lipoprotein receptor-1 (LOX1) [16].

### 2.1. Class B Scavenger Receptor: CD36

CD36 belongs to the class B scavenger receptors (SR) family [17]. SR comprise a structurally diverse group of membrane-bound proteins that are involved in cholesterol and lipoprotein metabolism [18]. SR are present on various cells including macrophages, monocytes, platelets, endothelial cells, smooth muscle cells and epithelial cells [18]. These receptors bind multiple ligands, including LDL, ox-LDL, acetylated LDL, modified high density lipoproteins (HDL), Gram-positive and Gram-negative bacteria, β-amyloid and advanced glycation end products (AGE) [19]. Because of SR’s ability to bind diverse ligands, they are involved in many physiological and pathological processes such as homeostasis, innate immunity and apoptotic cell clearance. Importantly, SR play a crucial role in the atherosclerotic plaque formation and progression [20].

CD36, an 88-kDa transmembrane glycoprotein receptor, is constitutively expressed on various cell types, including platelets [21]. Expression levels substantially vary in the human populations and have been linked to specific polymorphisms associated with the risk of myocardial infarction [22]. It was shown that ox-LDL binding to CD36 receptor triggers signaling pathways that activate platelets, inducing the expression of P-selectin and activation of integrin α_IIb_β_3_ (receptor for fibrinogen). Hence, ox-LDL binding to CD36 enables the formation of platelet-leukocyte complexes via P-selectin and cross-linking of the adjacent platelets via fibrinogen [15]. Further, the interaction between ox-LDL and CD36 was shown to trigger platelet hyperactivity in response to co-stimulation with ox-LDL and low-dose adenosine diphosphate (ADP) in a mouse model, as measured by flow cytometry [14,23].

**Figure 1:**
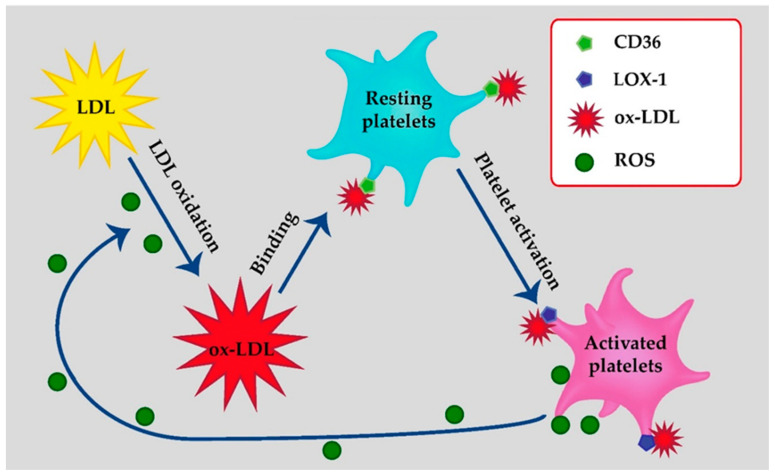
Interactions between low-density lipoproteins (LDL), oxidized LDL (ox)-LDL and platelets. LDL oxidation is assisted by reactive oxygen species (ROS). Ox-LDL bind to resting platelets by cluster of differentiation 36 (CD36), leading to platelet activation and expression of lectin-like oxidized low-density lipoprotein receptor-1 (LOX1) on platelets. Ox-LDL bind to activated platelets by LOX-1. As a result, activated platelets produce ROS, which results in a vicious circle of LDL oxidation and platelet activation.

The interaction between platelets and ox-LDL via CD36 is reciprocal. Not only ox-LDL activate platelets, but platelets bound to ox-LDL exert pro-atherogenic effects, including the release of atherosclerosis-promoting chemokines such as monocyte chemotactic protein-1 and interleukin 1β precursor [16,21]. Furthermore, platelets with internalized ox-LDL activate endothelial cells and inhibit endothelial regeneration [7]. Finally, in response to ox-LDL, platelets form aggregates with monocytes, and this aggregates facilitate the ox-LDL uptake by monocytes in a CD36-dependent manner [24]. Altogether, the interaction between ox-LDL and CD36 receptor on platelets triggers platelet activation and reciprocally facilitates ox-LDL formation, expanding endothelial injury and vascular inflammation. The interactions between LDL, ox-LDL and platelets are summarized in Figure 1.

### 2.2. Class E Scavenger Receptors: LOX-1

LOX-1 is a 50 kDa type 2 transmembrane glycoprotein that mediates the uptake of ox-LDL by endothelial cells and platelets [13,25]. Unlike CD36, which is constitutively expressed on platelets, LOX-1 is expressed in an activation-dependent manner [26]. Stimuli such as angiotensin II, cytokines, sheer stress, and AGE upregulate LOX-1 expression [27]. Clinical conditions including hypertension, diabetes, obesity, ischemia-reperfusion injury, heart failure and psychological stress have been shown to increase the LOX-1 expression as well [25].

LOX-1 effect on platelets resembles the effect of the well-established platelet activator ADP, which decreases the intracellular concentration of cyclic adenosine monophosphate (cAMP) [28]. Binding of ox-LDL to LOX-1 leads to activation of integrins α_IIb_β_3_ and α_2_β_1_, which are receptors for fibrinogen and collagen, respectively, crucial in thrombus formation [29]. The prothrombotic effect of LOX-1 activation has been confirmed by the in vitro experiments using the specific antibody against LOX-1, which inhibited binding of ox-LDL to platelets [13]. Anti-LOX-1 inhibited ADP-induced platelet aggregation in a time- and concentration-dependent manner, implying that LOX-1 acts as a co-agonist during activation of platelets with ADP [13]. Further, LOX-1 binds to activated platelets, facilitating cross-linking of platelets and thus stabilizing the thrombus [16]. Finally, it modulates plaque stability by affecting matrix metalloproteinases expression and activity, smooth muscle cell apoptosis and collagen content within the atherosclerotic plaques [25]. Altogether, LOX-1-mediated interactions between ox-LDL and platelets provide the molecular link between increased concentration of LDL-cholesterol and platelet hyperreactivity in patients with dyslipidemia [30].

## 3. Platelets as a Source of Oxidized LDL

Reactive oxygen species (ROS) are highly reactive molecules, generated in response to both endogenous and exogenous stimuli, which promotes protein, lipid and DNA damage. Increased local and systemic levels of ROS induce endothelial cell activation, dysfunction, apoptosis and impaired vasorelaxation, and contribute to atherosclerosis development and progression [12]. Traditionally, smooth muscle cells and macrophages are perceived as the main source of ROS and consequently, the main LDL-oxidating cells. Due to the imbalance between the ROS generation and antioxidant mechanisms in atherosclerosis, activated platelets also generate ROS and contribute to ox-LDL formation. On the other hand, increased level of ox-LDL reciprocally leads to the increased ROS production and dose-dependent increase in endothelial cells apoptosis, as demonstrated in human umbilical vein endothelial cells culture [31]. This results in a vicious circle of ROS generation, LDL oxidation and platelet activation (Figure 1) [32].

ROS are physiologically produced by a cell membrane enzymatic complex, nicotinamide adenine dinucleotide phosphate oxidase (NOX), and by mitochondria in the electron transport chain [32]. The pivotal role of ROS and mitochondria in platelet function has recently emerged [33]. Since ROS act as second messengers, they regulate platelet recruitment to the site of vascular injury, activation and aggregation [34]. The formation of platelet aggregates is associated with the burst of ROS, including hydrogen peroxide (H_2_O_2_) [35]. H_2_O_2_ induces platelet aggregation mediated by fibrinogen binding to integrin α_IIb_β_3,_ which is independent of ADP secreted from platelet dense granules [36]. H_2_O_2_ also enhances aggregation induced by phospholipid-derived arachidonate [33]. Altogether, ROS are a part of the machinery used by platelets to oxidize LDL [37].

NOX isoforms are the main sources of ROS in platelets, followed by cyclooxygenase (COX), xanthine oxidase (XO) and mitochondrial respiration [33]. In human platelets, both NOX1 and NOX2 contribute to ROS production. Both isoforms are inactive in resting platelets and become activated upon stimulation by agonists [38]. Ox-LDL-induced ROS, generation was markedly reduced by pharmacological inhibition of NOX2 and completely abolished in CD36- and NOX2-deficient mice [39]. Concurrently, patients with genetically determined NOX2 deficiency have both impaired platelet activation and inability to oxidize LDL, further confirming the mutual interactions between platelets and LDL [40].

## 4. Platelets as a Target of Oxidized LDL

Resting platelets take up ox-LDL at different concentrations [41]. When platelets are activated, internalization of ox-LDL is markedly enhanced [7]. Ox-LDL is stored intracellularly in the dense granules, along with ADP, ionized calcium and polyphosphates, all of which are potent platelet activators [7]. LDL alters platelet function by remodeling of phospholipids in the cell membrane and by insertion of phospholipids from circulating lipoproteins, thereby changing the composition of membrane phospholipids [42]. Thus, platelets are sensitized by LDL through the activation of a signal transduction pathways, as well as by the exchange of lipids [16].

Whereas native LDL does not affect platelet activation [7], binding of ox-LDL to platelet receptor CD36 leads to ROS production and subsequently triggers the loop of LDL oxidation and platelet activation, as showed by enhanced surface expression of specific platelet activation markers: P-selectin and glycoprotein (GP) VI [43]. Compared with native platelets, ox-LDL-laden platelets show increased adherence to collagen type I, which represents the major extracellular matrix compound of the damaged arterial wall [7].

Following ox-LDL intake and adherence to collagen, platelets lead to immune cells accumulation at the sites vascular injury [7]. Moreover, such platelets activate endothelial cells, which is reflected by increased intercellular adhesion molecule 1 (ICAM-1) expression [7]. The endothelial expression of ICAM-1 is increased in atherosclerotic cell cultures and in animal models of atherosclerosis. However, in vitro studies showed that ox-LDL may either activate or inhibit platelet function such as aggregation and β-thromboglobulin secretion [44], depending on the oxidized domain and the degree of oxidation [45]. These contradictions about platelet function inhibition and activation appear to stem from the heterogeneous nature of ox-LDL.

Altogether, following activation by ox-LDL, platelets seem to have increased prothrombotic, pro-inflammatory and proatherogenic properties. However, some controversies regarding the effect of different ox-LDL phenotypes on platelets remain to be elucidated.

## 5. Role of Activates Platelets and Ox-LDL in Atherosclerosis

The atherosclerotic process can either be clinically silent, or result in plaque expansion and vessel constriction, causing anginal symptoms. More dangerously, plaques may rupture, leading to a clinically manifest acute coronary syndrome, stroke or acute limb ischemia, depending on their location. There are various factors that determine plaque stability, including platelets function and their interaction with LDL.

Platelets are the first circulating blood cells that adhere to the sites of vascular injury [46]. Beyond their role in hemostasis and thrombosis, platelets play a critical role in atherogenesis [47]. Elevated plasma concentration of LDL is a well-established risk factor for atherosclerosis as well [48]. The interactions between platelets and ox-LDL affect (i) inflammatory cells activation, (ii) endothelial cells regeneration and (iii) foam cell generation, all of which contribute to atherosclerosis.

Both activated platelets and ox-LDL exert proinflammatory and procoagulant effects on several vascular cells (e.g., neutrophils, monocytes/macrophages, smooth muscle cells, endothelial cells, and platelets), thus supporting that atherosclerosis is a chronic inflammatory disease [44,49].

Activated platelets interact with endothelium and change the chemotactic and adhesive properties of endothelial cells, a critical initial step in atherogenesis [50]. In the presence of ox-LDL-laden platelets, endothelial cells are unable to generate an intact monolayer [7]. In this context, oxLDL-laden platelets promote vascular inflammation and inhibit vascular regeneration [7]. Ox-LDL were showed to impair adhesive, migratory and tube formation capacities of endothelial progenitor cells by an inhibitory effect on endothelial nitric oxide synthase, which was reversed by inhibition of the LOX-1 receptor [51].

Foam cell generation from macrophages with subsequent fatty streak formation plays a key role in the early atherogenesis [52]. Ox-LDL-laden platelets induce CD34^+^ progenitor cells transformation into macrophages or foam cells, depending on the microenvironment [53]. A two-fold increase in foam cell formation was observed in presence of oxLDL-positive platelets, compared with native LDL or native platelets [7]. In fact, reduction of platelet-induced foam cell generation is likely one of the mechanisms underlying the pleiotropic effects of statins [53].

**Figure 2:**
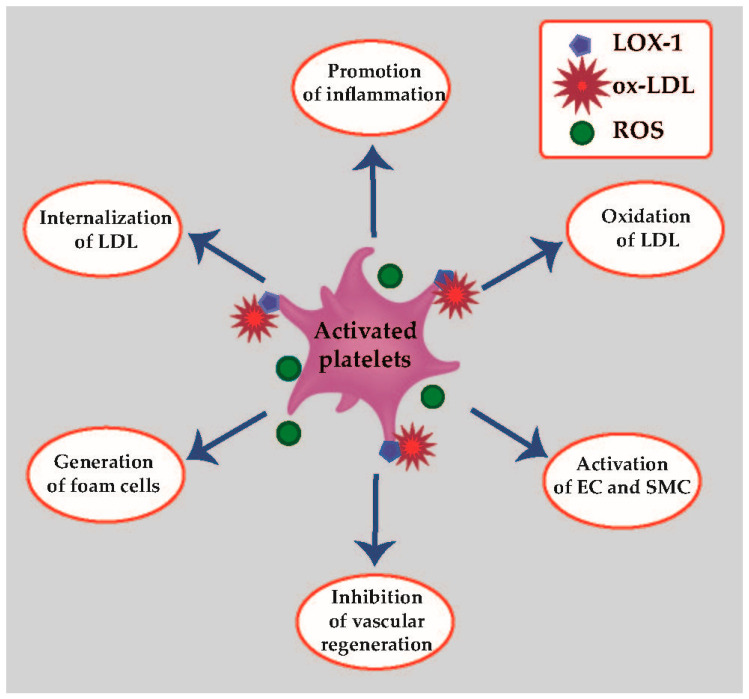
Role of activated platelets in atherosclerosis onset and development. EC—endothelial cells; LOX-1—lectin-like oxidized low-density lipoprotein receptor-1; ox-LDL—oxidized low-density lipoproteins; ROS—reactive oxygen species; SMC—smooth muscle cells.

The role of activated platelets and ox-LDL in atherosclerosis development and progression is presented in Figure 2.

## 6. Role of Platelet-Derived Extracellular Vesicles in Atherosclerosis

The mechanisms underlying platelet activation in atherosclerosis are still incompletely understood. Platelet-derived extracellular vesicles (PEVs) disseminate the procoagulant and proinflammatory effects of activated platelets, thus contributing to platelet interactions with other cells and with ox-LDL particles [54].

EVs are a diverse group of membrane-enclosed structures, released from cells to bloodstream in physiological and pathological conditions [55]. EVs act on target cells by delivering ligands and signaling complexes. For example, EVs transfer microRNAs and transcription factors that epigenetically reprogram the target cells [55]. EVs are shed by cells into the extracellular space during activation, maturation, proliferation, stress, aging, or apoptosis [56]. About 30% of all EVs present in normal plasma originated from platelets, as estimated by cryo-electron microscopy [57].

PEVs are secreted during platelets activation and share many functional features with activated platelets, this having a crucial role in platelet aggregation and thrombus formation [58]. For example, PEVs expose multiple prothrombotic molecules, including glycoproteins and phospholipids such as GP Ib, P-selectin and phosphatidylserine [59]. Further, PEVs activate platelets, as measured by the expression of platelet activation markers, secretion of soluble P-selectin and platelet aggregation [59]. Finally, PEVs and other extracellular vesicles accumulate in the lipid core of plaques and thrombi [60].

Similarly to platelets, PEVs may carry the CD36 receptor for LDL, resulting in generation of a PEV–LDL complexes [59]. It was shown that PEVs enhanced platelet ROS production in a CD36- and phosphatidylserine-dependent manner [59]. The formation of tissue factor-expressing EVs by a monocytic cell line was significantly enhanced in the presence of ox-LDL [61]. In addition, one clinical study comprising 41 patients with acute coronary syndrome (ACS), 22 patients with unstable angina (UA) and 20 healthy controls showed that elevated plasma concentrations of PEVs in ACS, compared to UA and controls. Among ACS patients, platelet EVs were higher in patients with a high level of ox-LDL, compared to those with low ox-LDL [61]. In addition, plasma concentrations of PEVs are affected both by antiplatelet drugs [62,63] and lipid-lowering drugs (statins) [64].

Altogether, the release of PEVs during platelet activation may not only have direct, but also indirect prothrombotic effect via interactions with ox-LDL, and both antiplatelet and lipid-lowering therapies may modulate PEV concentrations.

## 7. Clinical Implications

In patients with hypercholesterolemia, the abnormalities in platelet composition and function indicate that circulating lipoproteins in blood influence platelet properties [65,66]. Hypercholesterolemia is associated with increased platelet reactivity, which is reduced after administration of lipid-lowering therapies [67,68]. Platelets from hypercholesterolemic patients were found to have a higher potential to oxidize LDL and to promote LDL uptake by macrophages, compared to platelets from healthy donors [40]. Pharmacological modulation of platelet-LDL interaction via NOX-2, LOX-1 and CD-36 might potentially create new therapeutic opportunities to combat atherosclerosis.

### 7.1. NOX-2

NOX-2 dependent production of ROS is increased in platelets in presence of ox-LDL. ROS play an important role in platelet activation process. Inhibition of NOX2-derived ROS may represent a novel pathway to inhibit platelet aggregation and eventually thrombus growth [9]. At the moment, it remains an area of active research.

### 7.2. LOX-1

LOX-1 play an important role in ADP-induced activation of fibrinogen receptors, contributing to platelet aggregation and thrombus formation [29]. There are three main groups of drugs that can inhibit LOX-1.

#### 7.2.1. Antihypertensive Agents

Calcium channel blockers (CCB) and angiotensin II receptor blockers (ARB) decrease the progression of atherosclerosis and decrease the incidence of cardiovascular events [69]. CCB with proven inhibitory effects on LOX-1 include nifedipine, amlodipine and diltiazem [70,71,72]. ARB have also been postulated to inhibit LOX-1 expression [73].

#### 7.2.2. Hypoglycemic Agents

In chronic hyperglycemia, glucose reacts with proteins to form AGE, which on one hand are ligands for LOX-1, and on the other hand upregulate LOX-1 [25]. Sulfonylureas, biguanides and peroxisome proliferator-activated receptor-*γ* (PPAR-*γ*) agonists used in patients with diabetes mellitus were demonstrated to decrease LOX-1 expression independent of their hypoglycemic or insulin-sensitizing actions [25], thus decreasing the interactions via ox-LDL and LOX-1.

Sodium-glucose co-transporter-2 (SGLT2) inhibitors have recently emerged as a breakthrough therapy in diabetic patients due to substantial improvements in overall and cardiovascular outcomes [74]. SGLT2 inhibitors dapagliflozin and ipragliflozin were showed to suppress macrophage foam cell formation and normalize expression of LOX-1 receptor gene in a diabetic mouse model [75]. Moreover, in a recent prospective, randomized study including 126 patients with diabetic nephropathy, dapagliflozin decreased ROS generation and ox-LDL concentration, which was associated with improved outcomes during 1-year observation period [76]. Hence, the benefits of SGLT2 inhibitors seem to expand far beyond the pure anti-diabetic actions, with ox-LDL being one of their pharmacological targets, potentially underlying favorable clinical outcomes in patients treated with these drugs.

#### 7.2.3. Statins

Anti-inflammatory pleiotropic effects of statins are increasingly recognized as being responsible for the improved outcomes in atherosclerotic patients, including patients after myocardial infarction, stroke and those with peripheral artery disease [77]. Statins (i) reduce platelet-induced foam cell generation, (ii) promote formation of endothelial cells derived from CD34^+^ progenitor cells [53], and (iii) enhance plaque stability [25]. Statins may exert their beneficial effects via downregulation of the LOX-1 expression in vascular lesions [25]. Indeed, aspirin and pravastatin were showed to synergistically reduce LOX-1 expression on platelets [26]. Due to these pleiotropic properties, including modulation of the interactions between ox-LDL and platelets, statins are recommended in high cardiovascular risk patients regardless of the serum cholesterol level.

### 7.3. CD36

The presence of CD36 on platelets was confirmed by inhibition studies with antibodies directed against the ox-LDL binding domain of CD36 [78]. CD36 inhibition resulted in a reduction of ox-LDL binding to platelets [79]. Apolipoprotein E (apoE), in turn, plays an important role in lipid metabolism and clearance. In hyperlipidemic CD36^−/−^apoE^−/−^ mice, the absence of CD36 significantly protected the mice from the hyperlipidemia-related prothrombotic phenotype, when compared to hyperlipidemic apoE^−/−^ mice without CD36 deficiency [15]. Hence, inhibition of CD36 is a potentially promising therapeutic strategy in atherosclerosis. However, CD36 is present on a multiple type of cells, for example platelets, macrophages and monocytes, so that its selective inhibition is challenging.

Ox-LDL-CD36 combination acts via activation of extracellular signal-regulated kinase 5 (ERK5) kinase from mitogen-activated protein kinase (MAPK) kinase family [80]. ERK5 was recently identified in platelets and showed to function as a redox switch to promote maladaptive platelet signaling during myocardial infarction [81]. ERK5 pathway is a critical modulator of thrombosis in hyperlipidemic conditions. ERK5 is an effector to integrate the non-classic activation pathway of CD36 signaling into known platelet activation pathways [80]. ERK5 was activated in platelets by ox-LDL in a CD36-dependent manner and this activation requires generation of specific ROS. Pharmacological inhibition or targeted genetic interruption of CD36/ROS/ERK5 signaling pathway results in decreased platelet activation by ox-LDL ex vivo and ameliorated arterial thrombosis in a hyperlipidemic mouse model [80]. Hence, inhibition of this pathway might stop the reciprocal loop of platelet activation and LDL oxidation.

The PEV–CD36 complex also activates MAPK signals and contributes to platelet activation. In addition, PEVs aggravate oxidative stress in a CD36- and phosphatidylserine-dependent manner. It was observed that PEV concentrations are reduced in patients treated with P2Y12 antagonists, statins and eicosapentaenoic acid [82,83]. Hence, both PEVs and CD36 are potential targets to hamper the development and progression of atherosclerosis.

## 8. Ox-LDL as a Biomarker in Cardiovascular Disease

High concentration of ox-LDL in vascular wall may be associated with higher risk of future plaque rupture, potentially making ox-LDL an important marker of the ‘vulnerable plaque’, as well as offering opportunities for diagnostic imaging and the targeted delivery of therapeutic agents [84]. LDL concentrations are a well-established circulating biomarker reflecting general cardiovascular risk and a therapeutic target in patients with atherosclerosis. However, there is still scope for more specific biomarkers with pathophysiological relevance to improve the clinical risk prediction of cardiovascular events [85]. Elevated ox-LDL levels or PEVs concentrations may prove to be more causally-associated with cardiovascular events than LDL due to their central role in atherosclerotic plaque biology [48]. Considering this, oxLDL and PEVs may have a clinical potential to improve risk prediction beyond LDL. A recent meta-analysis revealed that elevated ox-LDL levels is associated with an increased risk of CVD events [86]. In turn, recent studies also showed that PEVs are promising biomarkers of platelet activation in atherosclerosis.

## 9. Conclusions

Platelets and ox-LDL interactions play a key role in atherosclerosis development and progression due to (i) the presence of LDL-binding sites on platelets, (ii) generation of ox-LDL by platelets and (iii) targeting platelet by ox-LDL. The ability to understand and modulate mechanisms governing these interactions may offer new treatment strategies for atherosclerosis prevention. At the moment, the interactions between ox-LDL and platelets may be alleviated using three major groups of drugs: antihypertensive agents, antihyperglycemic agents and statins. However, since CD36 and LOX-1 are molecules in atherosclerosis development and progression, their pharmacological inhibition would be an exciting and promising avenue in developing therapeutic agents to alleviate or even reverse the atherosclerotic plaque formation. Modulation of proinflammatory and prothrombotic properties of PEVs-ox-LDL interactions are another potential future therapeutic option. Further research on the interactions between platelets and ox-LDL is urgently needed to optimize primary and secondary prevention of atherosclerosis.

## Abbreviations

ACS	acute coronary syndrome
ADP	adenosine diphosphate
AGE	advanced glycation end products
apoE	apolipoprotein E
ARB	angiotensin II receptor blockers
cAMP	cyclic adenosine monophosphate
CCB	calcium channel blockers
CD	cluster of differentiation
COX	cyclooxygenase
EC	Endothelial cells
ERK5	extracellular signal-regulated kinase 5
EVs	extracellular vesicles
GP	glycoprotein
H_2_O_2_	hydrogen peroxide
HDL	high density lipoproteins
ICAM-1	intercellular adhesion molecule 1
LDL	low-density lipoproteins
LOX1	lectin-like oxidized low-density lipoprotein receptor-1
MAPK	mitogen-activated protein kinase
NO	nitric oxide
NOX	nicotinamide adenine dinucleotide phosphate oxidase
oxLDL	oxidized low-density lipoproteins
PEVs	Platelet-derived extracellular vesicles
PPAR-*γ*	peroxisome proliferator-activated receptor-*γ*
ROS	reactive oxygen species
SGLT2	sodium-glucose co-transporter-2
SMC	smooth muscle cells
SR	scavenger receptors
UA	unstable angina
XO	xanthine oxidase

## References

[1] Barquera S., Pedroza-Tobias A., Medina C., Hernández-Barrera L., Bibbins-Domingo K., Lozano R., Moran A.E. (2015). Global overview of the epidemiology of atherosclerotic cardiovascular disease. Arch. Med. Res..

[2] Mozaffarian D., Benjamin E.J., Go A.S., Arnett D.K., Blaha M.J., Cushman M., De Ferranti S., Després J.-P., Fullerton H.J., Howard V.J. (2015). Executive summary: Heart disease and stroke statistics—2015 update: A report from the American Heart Association. Circulation.

[3] Alfarisi H.A.H., Mohamed Z.B.H., Ibrahim M. (2020). Bin Basic pathogenic mechanisms of atherosclerosis. Egypt. J. Basic Appl. Sci..

[4] Davignon J., Ganz P. (2004). Role of endothelial dysfunction in atherosclerosis. Circulation.

[5] Badimón L., Vilahur G., Padró T. (2009). Lipoproteinas, plaquetas y aterotrombosis. Rev. Esp. Cardiol..

[6] Fuentes Q.E., Fuentes Q.F., Andrés V., Pello O.M., de Mora J.F., Palomo G.I. (2013). Role of platelets as mediators that link inflammation and thrombosis in atherosclerosis. Platelets.

[7] Daub K., Seizer P., Stellos K., Krämer B.F., Bigalke B., Schaller M., Fateh-Moghadam S., Gawaz M., Lindemann S. (2010). Oxidized LDL-activated platelets induce vascular inflammation. Semin. Thromb. Hemost..

[8] Pirillo A., Norata G.D., Catapano A.L. (2013). LOX-1, OxLDL, and atherosclerosis. Mediat. Inflamm..

[9] Carnevale R., Bartimoccia S., Nocella C., Di Santo S., Loffredo L., Illuminati G., Lombardi E., Boz V., Del Ben M., De Marco L. (2014). LDL oxidation by platelets propagates platelet activation via an oxidative stress-mediated mechanism. Atherosclerosis.

[10] Levitan I., Volkov S., Subbaiah P.V. (2010). Oxidized LDL: Diversity, patterns of recognition, and pathophysiology. Antioxid. Redox Signal..

[11] Garraud O., Cognasse F. (2015). Are platelets cells? And if yes, are they immune cells?. Front. Immunol..

[12] Parthasarathy S., Raghavamenon A., Garelnabi M.O., Santanam N. (2010). Oxidized low-density lipoprotein. Free Radicals and Antioxidant Protocols.

[13] Chen M., Kakutani M., Naruko T., Ueda M., Narumiya S., Masaki T., Sawamura T. (2001). Activation-dependent surface expression of LOX-1 in human platelets. Biochem. Biophys. Res. Commun..

[14] Chen K., Febbraio M., Li W., Silverstein R.L. (2008). A specific CD36-dependent signaling pathway is required for platelet activation by oxidized low-density lipoprotein. Circ. Res..

[15] Podrez E.A., Byzova T.V., Febbraio M., Salomon R.G., Ma Y., Valiyaveettil M., Poliakov E., Sun M., Finton P.J., Curtis B.R. (2007). Platelet CD36 links hyperlipidemia, oxidant stress and a prothrombotic phenotype. Nat. Med..

[16] Siegel-Axel D., Daub K., Seizer P., Lindemann S., Gawaz M. (2008). Platelet lipoprotein interplay: Trigger of foam cell formation and driver of atherosclerosis. Cardiovasc. Res..

[17] Calvo D., Dopazo J., Vega M.A. (1995). The CD36, CLA-1 (CD36L1), and LIMPII (CD36L2) gene family: Cellular distribution, chromosomal location, and genetic evolution. Genomics.

[18] Ashraf M.Z., Sahu A. (2012). Scavenger receptors: A key player in cardiovascular diseases. Biomol. Concepts.

[19] Plüddemann A., Neyen C., Gordon S. (2007). Macrophage scavenger receptors and host-derived ligands. Methods.

[20] Krieger M. (1997). The other side of scavenger receptors: Pattern recognition for host defense. Curr. Opin. Lipidol..

[21] Park Y.M. (2014). CD36, a scavenger receptor implicated in atherosclerosis. Exp. Mol. Med..

[22] Ghosh A., Murugesan G., Chen K., Zhang L., Wang Q., Febbraio M., Anselmo R.M., Marchant K., Barnard J., Silverstein R.L. (2011). Platelet CD36 surface expression levels affect functional responses to oxidized LDL and are associated with inheritance of specific genetic polymorphisms. Blood.

[23] Chen K., Li W., Major J., Rahaman S.O., Febbraio M., Silverstein R.L. (2011). Vav guanine nucleotide exchange factors link hyperlipidemia and a prothrombotic state. Blood J. Am. Soc. Hematol..

[24] Badrnya S., Schrottmaier W.C., Kral J.B., Yaiw K.-C., Volf I., Schabbauer G., Söderberg-Nauclér C., Assinger A. (2014). Platelets Mediate Oxidized Low-Density Lipoprotein--Induced Monocyte Extravasation and Foam Cell Formation. Arterioscler. Thromb. Vasc. Biol..

[25] Xu S., Ogura S., Chen J., Little P.J., Moss J., Liu P. (2013). LOX-1 in atherosclerosis: Biological functions and pharmacological modifiers. Cell. Mol. Life Sci..

[26] Kattoor A.J., Kanuri S.H., Mehta J.L. (2019). Role of Ox-LDL and LOX-1 in Atherogenesis. Curr. Med. Chem..

[27] Kattoor A.J., Goel A., Mehta J.L. (2019). LOX-1: Regulation, signaling and its role in atherosclerosis. Antioxidants.

[28] Mattaliano M.D., Huard C., Cao W., Hill A.A., Zhong W., Martinez R.V., Harnish D.C., Paulsen J.E., Shih H.H. (2009). LOX-1-dependent transcriptional regulation in response to oxidized LDL treatment of human aortic endothelial cells. Am. J. Physiol. Physiol..

[29] Marwali M.R., Hu C.-P., Mohandas B., Dandapat A., Deonikar P., Chen J., Cawich I., Sawamura T., Kavdia M., Mehta J.L. (2007). Modulation of ADP-induced platelet activation by aspirin and pravastatin: Role of lectin-like oxidized low-density lipoprotein receptor-1, nitric oxide, oxidative stress, and inside-out integrin signaling. J. Pharmacol. Exp. Ther..

[30] Jäger B., Piackova E., Haller P.M., Andric T., Kahl B., Christ G., Geppert A., Wojta J., Huber K. (2019). Increased platelet reactivity in dyslipidemic patients with coronary artery disease on dual anti-platelet therapy. Arch. Med. Sci. AMS.

[31] Chen X., Xun K., Wu Q., Zhang T., Shi J., Du G. (2007). Oxidized low density lipoprotein receptor-1 mediates oxidized low density lipoprotein-induced apoptosis in human umbilical vein endothelial cells: Role of reactive oxygen species. Vascul. Pharmacol..

[32] Bae Y.S., Oh H., Rhee S.G., Do Yoo Y. (2011). Regulation of reactive oxygen species generation in cell signaling. Mol. Cells.

[33] Wachowicz B., Olas B., Zbikowska H.M., Buczyński A. (2002). Generation of reactive oxygen species in blood platelets. Platelets.

[34] Qiao J., Arthur J.F., Gardiner E.E., Andrews R.K., Zeng L., Xu K. (2018). Regulation of platelet activation and thrombus formation by reactive oxygen species. Redox Biol..

[35] Hedin H.L.M., Fowler C.J. (1999). Further studies of the effects of diamide and hydrogen peroxide on calcium signaling in the human platelet. Methods Find. Exp. Clin. Pharmacol..

[36] Pignatelli P., Pulcinelli F.M., Lenti L., Paolo Gazzaniga P., Violi F. (1998). Hydrogen peroxide is involved in collagen-induced platelet activation. Blood J. Am. Soc. Hematol..

[37] Masselli E., Pozzi G., Vaccarezza M., Mirandola P., Galli D., Vitale M., Carubbi C., Gobbi G. (2020). ROS in platelet biology: Functional aspects and methodological insights. Int. J. Mol. Sci..

[38] Fuentes E., Gibbins J.M., Holbrook L.M., Palomo I. (2018). NADPH oxidase 2 (NOX_2_): A key target of oxidative stress-mediated platelet activation and thrombosis. Trends Cardiovasc. Med..

[39] Pignatelli P., Sanguigni V., Lenti L., Ferro D., Finocchi A., Rossi P., Violi F. (2004). gp91phox-dependent expression of platelet CD40 ligand. Circulation.

[40] Carnevale R., Pignatelli P., Lend L., Buchetti B., Sanguigni V., Di Santo S., Violi F. (2007). LDL are oxidatively modified by platelets via GP91phox and accumulate in human monocytesLDL are oxidatively modified by platelets via GP91phox and accumulate in human monocytes. FASEB J..

[41] Gawaz M., Brand K., Dickfeld T., Pogatsa-Murray G., Page S., Bogner C., Koch W., Schömig A., Neumann F.-J. (2000). Platelets induce alterations of chemotactic and adhesive properties of endothelial cells mediated through an interleukin-1-dependent mechanism. Implications for atherogenesis. Atherosclerosis.

[42] Engelmann B., Kögl C., Kulschar R., Schaipp B. (1996). Transfer of phosphatidylcholine, phosphatidylethanolamine and sphingomyelin from low-and high-density lipoprotein to human platelets. Biochem. J..

[43] Marcus A.J., Silk S.T., Safier L.B., Ullman H.L. (1977). Superoxide production and reducing activity in human platelets. J. Clin. Investig..

[44] Miyazaki A., Uehara T., Usami Y., Ishimine N., Sugano M., Tozuka M. (2020). Highly oxidized low-density lipoprotein does not facilitate platelet aggregation. J. Int. Med. Res..

[45] Resmi K.R., Krishnan L.K. (2002). Protease action and generation of β-thromboglobulin-like protein followed by platelet activation. Thromb. Res..

[46] Ruggeri Z.M. (2002). Platelets in atherothrombosis. Nat. Med..

[47] Gawaz M., Langer H., May A.E. (2005). Platelets in inflammation and atherogenesis. J. Clin. Investig..

[48] Hartley A., Haskard D., Khamis R. (2019). Oxidized LDL and anti-oxidized LDL antibodies in atherosclerosis—Novel insights and future directions in diagnosis and therapy. Trends Cardiovasc. Med..

[49] Di Pietro N., Formoso G., Pandolfi A. (2016). Physiology and pathophysiology of oxLDL uptake by vascular wall cells in atherosclerosis. Vascul. Pharmacol..

[50] Gawaz M. (2004). Role of platelets in coronary thrombosis and reperfusion of ischemic myocardium. Cardiovasc. Res..

[51] Ma F.X., Zhou B., Chen Z., Ren Q., Lu S.H., Sawamura T., Han Z.C. (2006). Oxidized low density lipoprotein impairs endothelial progenitor cells by regulation of endothelial nitric oxide synthase. J. Lipid Res..

[52] Shashkin P., Dragulev B., Ley K. (2005). Macrophage differentiation to foam cells. Curr. Pharm. Des..

[53] Daub K., Langer H., Seizer P., Stellos K., May A.E., Goyal P., Bigalke B., Schönberger T., Geisler T., Siegel-Axel D. (2006). Platelets induce differentiation of human CD34+ progenitor cells into foam cells and endothelial cells. FASEB J..

[54] Gasecka A., Nieuwland R., Siljander P.R.-M. (2019). Platelet-derived extracellular vesicles. Platelets.

[55] Żmigrodzka M., Witkowska-Piłaszewicz O., Winnicka A. (2020). Platelets Extracellular Vesicles as Regulators of Cancer Progression—An Updated Perspective. Int. J. Mol. Sci..

[56] Stahl P.D., Raposo G. (2019). Extracellular vesicles: Exosomes and microvesicles, integrators of homeostasis. Physiology.

[57] Hunter M.P., Ismail N., Zhang X., Aguda B.D., Lee E.J., Yu L., Xiao T., Schafer J., Lee M.-L.T., Schmittgen T.D. (2008). Detection of microRNA expression in human peripheral blood microvesicles. PLoS ONE.

[58] van der Pol E., Harrison P. (2017). From platelet dust to gold dust: Physiological importance and detection of platelet microvesicles. Platelets.

[59] Wang H., Wang Z.-H., Kong J., Yang M.-Y., Jiang G.-H., Wang X.-P., Zhong M., Zhang Y., Deng J.-T., Zhang W. (2012). Oxidized low-density lipoprotein-dependent platelet-derived microvesicles trigger procoagulant effects and amplify oxidative stress. Mol. Med..

[60] Leroyer A.S., Tedgui A., Boulanger C.M. (2008). Role of microparticles in atherothrombosis. J. Intern. Med..

[61] Matsumoto N., Nomura S., Kamihata H., Kimura Y., Iwasaka T. (2004). Increased level of oxidized LDL-dependent monocytederived microparticles in acute coronary syndrome. Thromb. Haemost..

[62] Gasecka A., Nieuwland R., Budnik M., Dignat-George F., Eyileten C., Harrison P., Lacroix R., Leroyer A., Opolski G., Pluta K. (2020). Ticagrelor attenuates the increase of extracellular vesicle concentrations in plasma after acute myocardial infarction compared to clopidogrel. J. Thromb. Haemost..

[63] Gasecka A., Nieuwland R., van der Pol E., Hajji N., Ćwiek A., Pluta K., Konwerski Michałand Filipiak K.J. (2019). P2Y12 antagonist ticagrelor inhibits the release of procoagulant extracellular vesicles from activated platelets: Preliminary results. Cardiol. J..

[64] Suades R., Padro T., Alonso R., Mata P., Badimon L. (2013). Lipid-lowering therapy with statins reduces microparticle shedding from endothelium, platelets and inflammatory cells. Thromb. Haemost..

[65] Ferroni P., Basili S., Santilli F., Davi G. (2006). Low-density lipoprotein-lowering medication and platelet function. Pathophysiol. Haemost. Thromb..

[66] Elisaf M., Karabina S.A.P., Bairaktari E., Goudevenos J.A., Siamopoulos K.C., Tselepis A.D. (1999). Increased platelet reactivity to the aggregatory effect of platelet activating factor, in vitro, in patients with heterozygous familial hypercholesterolaemia. Platelets.

[67] Aviram M., Brook J.G. (1987). Platelet activation by plasma lipoproteins. Prog. Cardiovasc. Dis..

[68] Betteridge D.J., Cooper M.B., Saggerson E.D., Prichard B.N.C., Tan K.C.B., Ling E., Barbera G., McCarthy S., Smith C.C.T. (1994). Platelet function in patients with hypercholesterolaemia. Eur. J. Clin. Investig..

[69] Kang B.-Y., Wang W., Palade P., Sharma S.G., Mehta J.L. (2009). Cardiac hypertrophy during hypercholesterolemia and its amelioration with rosuvastatin and amlodipine. J. Cardiovasc. Pharmacol..

[70] Sugano M., Tsuchida K., Makino N. (2002). Nifedipine prevents apoptosis of endothelial cells induced by oxidized low-density lipoproteins. J. Cardiovasc. Pharmacol..

[71] Zhou M.-S., Jaimes E.A., Raij L. (2004). Inhibition of oxidative stress and improvement of endothelial function by amlodipine in angiotensin II-infused rats. Am. J. Hypertens..

[72] Rudijanto A. (2010). Calcium channel blocker (diltiazem) inhibits apoptosis of vascular smooth muscle cell exposed to high glucose concentration through lectin-like oxidized low density lipoprotein receptor-1 (LOX-1) pathway. Acta Med. Indones.

[73] Li D., Saldeen T., Romeo F., Mehta J.L. (2000). Oxidized LDL upregulates angiotensin II type 1 receptor expression in cultured human coronary artery endothelial cells: The potential role of transcription factor NF-κB. Circulation.

[74] David L. (2020). 2019 ESC Guidelines on diabetes, pre-diabetes, and cardiovascular diseases developed in collaboration with the EASD. Eur. Heart J..

[75] Terasaki M., Hiromura M., Mori Y., Kohashi K., Nagashima M., Kushima H., Watanabe T., Hirano T. (2015). Amelioration of hyperglycemia with a sodium-glucose cotransporter 2 inhibitor prevents macrophage-driven atherosclerosis through macrophage foam cell formation suppression in type 1 and type 2 diabetic mice. PLoS ONE.

[76] Jian X., Yang Q.L., Xiao S., Jing Z., Hu S.D. (2018). The effects of a sodium-glucose cotransporter 2 inhibitor on diabetic nephropathy and serum oxidized low-density lipoprotein levels. Eur. Rev. Med. Pharmacol. Sci.

[77] Schönbeck U., Libby P. (2004). Inflammation, immunity, and HMG-CoA reductase inhibitors: Statins as antiinflammatory agents?. Circulation.

[78] Asch A.S., Barnwell J., Silverstein R.L., Nachman R.L. (1987). Isolation of the thrombospondin membrane receptor. J. Clin. Investig..

[79] Volf I., Moeslinger T., Cooper J., Schmid W., Koller E. (1999). Human platelets exclusively bind oxidized low density lipoprotein showing no specificity for acetylated low density lipoprotein. FEBS Lett..

[80] Yang M., Cooley B.C., Li W., Chen Y., Vasquez-Vivar J., Scoggins N.O., Cameron S.J., Morrell C.N., Silverstein R.L. (2017). Platelet CD36 promotes thrombosis by activating redox sensor ERK5 in hyperlipidemic conditions. Blood.

[81] Cameron S.J., Ture S.K., Mickelsen D., Chakrabarti E., Modjeski K.L., McNitt S., Seaberry M., Field D.J., Le N.-T., Abe J. (2015). Platelet extracellular regulated protein kinase 5 is a redox switch and triggers maladaptive platelet responses and myocardial infarct expansion. Circulation.

[82] Nomura S., Inami N., Shouzu A., Omoto S., Kimura Y., Takahashi N., Tanaka A., Urase F., Maeda Y., Ohtani H. (2009). The effects of pitavastatin, eicosapentaenoic acid and combined therapy on platelet-derived microparticles and adiponectin in hyperlipidemic, diabetic patients. Platelets.

[83] Nomura S., Taniura T., Shouzu A., Omoto S., Suzuki M., Okuda Y., Ito T. (2018). Effects of sarpogrelate, eicosapentaenoic acid and pitavastatin on arterioslcerosis obliterans-related biomarkers in patients with type 2 diabetes (SAREPITASO study). Vasc. Health Risk Manag..

[84] Van Dijk R.A., Kolodgie F., Ravandi A., Leibundgut G., Hu P.P., Prasad A., Mahmud E., Dennis E., Curtiss L.K., Witztum J.L. (2012). Differential expression of oxidation-specific epitopes and apolipoprotein (a) in progressing and ruptured human coronary and carotid atherosclerotic lesions. J. Lipid Res..

[85] Sandhu P.K., Musaad S.M.A., Remaley A.T., Buehler S.S., Strider S., Derzon J.H., Vesper H.W., Ranne A., Shaw C.S., Christenson R.H. (2016). Lipoprotein biomarkers and risk of cardiovascular disease: A laboratory medicine best practices (LMBP) systematic review. J. Appl. Lab. Med..

[86] Gao S., Zhao D., Wang M., Zhao F., Han X., Qi Y., Liu J. (2017). Association between circulating oxidized LDL and atherosclerotic cardiovascular disease: A meta-analysis of observational studies. Can. J. Cardiol..

